# Supplementary material for: Urinary Viral Spectrum in Patients with Interstitial Cystitis/Bladder Pain Syndrome and the Clinical Efficacy of Valacyclovir Treatment
Source: Biomedicines. 2024 Feb 26;12(3):522. doi: 10.3390/biomedicines12030522 (PMC10967905; doi:10.3390/biomedicines12030522)
Supplement: Supplementary file 1 [file biomedicines-12-00522-s001.zip › Supplementary material study protocol.pdf]

# Urinary viral spectrum in patients with interstitial cystitis/bladder pain syndrome and the clinical efficacy of valacyclovir treatment

## Supplementary Data----Study protocol

**The role of Epstein Barr virus activation in bladder inflammation of patients with interstitial cystitis/bladder pain syndrome**

Version No.1.0, January 10$^{th}$, 2021

**Protocol No.:** MOST Taiwan 108-2314-B-303 -003, 109-2314-B-303 -024, and 111-2314-B-303-033-MY3
**Registration No.:** ClinicalTrials.gov Identifier: NCT050944145
**Trial phase:** Phase I
**Principal Investigator:** Jia-Fong Jhang
**Clinical trial institution:** Hualien Tzu Chi Hospital
**Compliance Statement:** This trial will be conducted in strict accordance with Good Clinical Practice
(GCP)

**Introduction:**

The treatment of IC/BPS is always a great challenge in urologists. Most treatments for IC/BPS could relief symptom in the patients, however, cure for IC/BPS is rare. Since our study revealed EBV infection might be a possible etiology of IC/BPS, anti-virus medications might have potential to treat or relief symptoms in the patients with IC/BPS. Acyclovir and valacyclovir are nucleoside analogs that selectively inhibit the replication of herpes simplex virus, and it has been widely used to treat systemic or solid organ EBV infection since 25 years ago. [1-4] Oral acyclovir and valacyclovir could reduce EBV shedding in T lymphocyte in the oropharynx during infectious mononucleosis. For the EBV-related malignancies, which existed EBV latency infection lymphocytes in the tissue, antiviral drugs also have potential to destroy infected cells. [5] The most common adverse effects of antivirus medications for herpesvirus are headache, nausea, diarrhea. However, the proportion of adverse effects of oral acyclovir and valacyclovir are just similar to the placebo group. [6] Hence, using antivirus drugs for IC/BPS patients with evidence of EBV infection in bladder should be a safe and reasonable treatment.

**Material and methods:**

We would like to conduct a prospective study to evaluate to therapeutic effect of oral valacyclovir for the patients with IC/BPS. Patients with IC/BPS would be enrolled by Dr. Kuo and Dr. Jhang in their urology clinic. The diagnosis of IC/BPS was made through following the clinical symptom index of the American Urological Association guideline: "An unpleasant sensation (pain, pressure, discomfort) perceived to be related to the urinary bladder, associated with lower urinary tract symptoms of more than six weeks duration, in the absence of infection or other identifiable causes."[7]

**Patients inclusion criteria:**

1. Patients who were diagnosed with IC/BPS for more than 1 years, including the patients with Hunner's lesion and without Hunner's lesion.
2. Patients with IC/BPS and refractory to oral medications and intravesical installation or injection therapies (including hyaluronic acid installation and botulinum toxin injection)
3. Patients are will to receive valacyclovir treatment, could be followed for 1 month, and could provide urine specimens

**Patients exclusion criteria:**

1. Patients with neurogenic voiding dysfunction
2. Patients who had used medication which might induce bladder inflammation, such as ketamine and cyclophosphamide
3. Patients with bladder outlet obstruction
4. Patients with urolithiasis
5. Patients with history of any cancer
6. Patients who had previously used valacyclovir

At first, the enrolled patients would be investigated for urinary analysis and culture to rule out current bacteriuria. The enrolled patients would receive a comprehensive medical history review. A pregnancy test would be performed for women of childbearing potential. The patients would be asked to provide urine sample to urinary virus investigation before the treatment. The IC/BPS patients would be investigated for baseline clinical symptoms

questionnaire including visual analog scale (VAS) score for pain, quality of life score (QoL) based on urinary symptoms, Interstitial Cystitis Symptoms Index (ICSI), and Interstitial Cystitis Problem Index (ICPI), and OSS=ICSI+ICPI. The parameters in the VUDS report also would be record. The Informed consent would be obtained from all individual participants before the intervention. Due to higher bioavailability, we would use valacyclovir in this study. The dose of valacyclovir we use is according to previous valacyclovir study for genital HSV infection. [8] According previous valacyclovir study for chronic recurrence herpesvirus infection disease, long-term therapy of oral valacyclovir < 1000mg/day should be effective and safe; the safety profiles of valacyclovir and placebo were similar. [9] All of our patients would receive open-label oral valacyclovir 500 mg twice per day for 4 weeks.

We estimate to enrol IC/BPS patients with evidence of EBV infection in bladder into this study. After the beginning of valacyclovir therapy, the patients would receive a telephone interview to evaluated current symptoms changes at first and second weeks. The overall improvement of current therapy would be assessed with Global Response Assessment (GRA) (3: symptoms free, 2: >50% symptoms improvement, 1: 25-50% symptoms improvement, 0: 0-25% symptoms improvement, -1: symptoms worse). The GRA, VAS, and any adverse effect would be investigated in the telephone interview. After the full 4 weeks treatment, the patients would be asked to back to our clinic. All symptoms questionnaire (GRA, VAS, QoL, ICPI, ICSI, and OSS) and adverse effect would be evaluated again. The patients would be asked to provide urine sample again. The primary endpoint of this study is the VAS pain score changes at 4 weeks. Using qPCR, the urine EBV presence and amount also would be compare between baseline and post-treatment. The secondary endpoints include the changes of VAS, VAS, QoL, ICPI, ICSI, and OSS between baseline and end of the study. The following figure showed the study flow diagram.

We used G*Power 3.1.9.2 to calculate the sample size needed. For evaluating the change of VAS pain score of intervention in this before-after study, we set effect size of 0.6, $\alpha$ of 0.05, power(1-$\beta$) of 0.80, and two-sided test then got the estimated sample size 24. For estimating dropout rate 15%, we enrolled 28 patients with IC/BPS for the treatment. Finally all patients completed the study and were included into the final analysis

**Figure S1.** The study flow diagram and follow-up timing

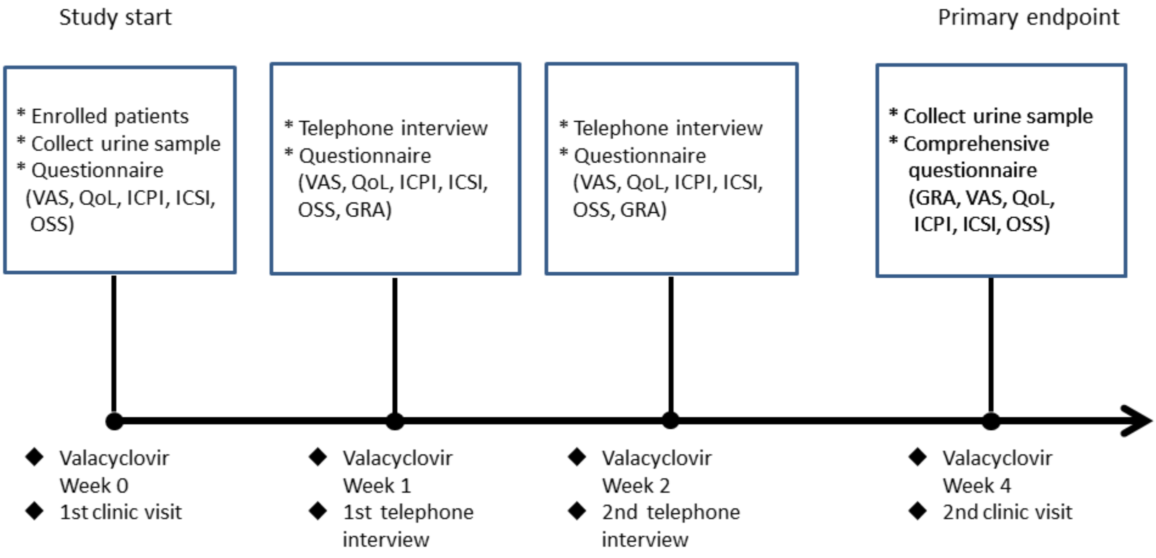

### Urinary virus and inflammatory cytokine investigation

Urine samples of 50 mL would be collected at baseline and 4 weeks after valacyclovir treatment. The samples were placed immediately on ice and then centrifuged at 1800× g for 10 minutes at 4°C. The supernatant was preserved in a freezer at −80°C. Before further analysis, the frozen urine samples were centrifuged at 12,000× g for 15 minutes at 4°C, and the supernatants were used for subsequent experiments. The centrifuged supernatants (1 mL) were sent to the Medical Laboratory Department in Hualien Tzu Chi Hospital for urinary virus investigation. The urinary virus targets were EBV, BKV, JCV, herpes simplex virus (HSV), varicella-zoster virus (VZV), and cytomegalovirus (CMV). The EBV DNA (BNRF1) detection by PCR was performed using a commercialized kit (Primerdesign, Chandler's Ford, UK). The BKV and JCV DNA detection was also performed with commercialized kits from Primerdesign. The urinary VZV and HSV were investigated with another commercialized kit (LightMix, Roche, Basel, Switzerland, Cat.-No. 40-0358-96 and Cat.-No. 40-0562-32, respectively).

### Study approval

This study was approved by Institutional Review Board and Ethics Committee of Hualien Tzu Chi Hospital (IRB number 108-45-A). The IRB for this study was approved on July 1st, 2019. All patients were informed about the rationale, risk, and procedures.

### Statistical analysis

The clinical and laboratory results for each group would be compared using nonparametric methods, including the Mann–Whitney U test for 2 nonpaired groups and the Wilcoxon matched-pair signed-rank test for 2 paired groups. A p value of <0.05 indicate a significant difference. All statistical analyses would be performed using GraphPad Prism 8 (Boston, MA, USA).

### Reference


1. Andersson, J., Skoldenberg, B., Ernberg, I. et al.: Acyclovir treatment in primary Epstein-Barr virus infection. A double-blind placebo-controlled study. Scand J Infect Dis Suppl, **47:** 107, 1985
2. Pagano, J. S., Sixbey, J. W., Lin, J. C.: Acyclovir and Epstein-Barr virus infection. J Antimicrob Chemother, **12 Suppl B:** 113, 1983
3. Sullivan, J. L., Byron, K. S., Brewster, F. E. et al.: Treatment of life-threatening Epstein-Barr virus infection with acyclovir. Am J Med, **73:** 262, 1982
4. Pagano, J. S., Whitehurst, C. B., Andrei, G.: Antiviral Drugs for EBV. Cancers (Basel), **10**, 2018
5. Gershburg, E., Pagano, J. S.: Epstein-Barr virus infections: prospects for treatment. J Antimicrob Chemother, **56:** 277, 2005
6. Tyring, S. K., Douglas, J. M., Jr., Corey, L. et al.: A randomized, placebo-controlled comparison of oral valacyclovir and acyclovir in immunocompetent patients with recurrent genital herpes infections. The Valaciclovir International Study Group. Arch Dermatol, **134:** 185, 1998
7. Hanno, P. M., Erickson, D., Moldwin, R. et al.: Diagnosis and treatment of interstitial cystitis/bladder pain syndrome: AUA guideline amendment. J Urol, **193:** 1545, 2015
8. Leone, P. A., Trottier, S., Miller, J. M.: Valacyclovir for episodic treatment of genital herpes: a shorter 3-day treatment course compared with 5-day treatment. Clin Infect Dis, **34:** 958, 2002
9. Tyring, S. K., Baker, D., Snowden, W.: Valacyclovir for herpes simplex virus infection: long-term safety and sustained efficacy after 20 years' experience with acyclovir. J Infect Dis, **186 Suppl 1:** S40, 2002

STANDARD PROTOCOL ITEMS: RECOMMENDATIONS FOR INTERVENTIONAL TRIALS

SPIRIT 2013 Checklist: Recommended items to address in a clinical trial protocol and related documents*

| Section/item | Item No | Description |
|---|---|---|
| **Administrative information** | | |
| Title | 1 | Using Oral Valacyclovir to Treat Patients With Refractory IC/BPS (VARIC) |
| Trial registration | 2a | ClinicalTrials.gov Identifier: NCT05094414 |
| Protocol version | 3 | January 10th, 2021, Version 1 |
| Funding | 4 | Ministry of Science and Technology, Taiwan and Hualien Tzu Chi General Hospital |
| Roles and responsibilities | 5a | Jia-Fong Jhang, Department of Urology, Hualien Tzu Chi General Hospital, Taiwan; study design and patients recruitment |
| | 5b | Ministry of Science and Technology, Taiwan: tlyang@most.gov.tw Hualien Tzu Chi General Hospital, Taiwan: hlmweb@tzuchi.com.tw. |
| | 5c | The funders do not have any role in study design; collection, management, analysis, and interpretation of data; writing of the report; or the decision to submit the report for publication. |
| | 5d | The principal investigator Jia-Fong Jhang led the research team for this clinical trial and is responsible for results. The data would be overseen by Research Ethics Committee of Hualien Tzu Chi General Hospital. |
| **Introduction** | | |
| Background and rationale | 6a | Interstitial cystitis/bladder pain syndrome (IC/BPS) is a collective term referring to disorders which is characterized by lower urinary tract symptoms, including bladder pain/discomfort, frequent urination without evidence of bacterial infection. The etiology of IC/BPS is still uncertain, and most current treatment for IC/BPS are only symptoms control. Our previous study revealed Epstein-Barr virus (EBV) infection presented in the IC/BPS bladders and involved the pathogenesis (J Urol. 2018;200:590-596). Hence, using anti-viral medication valacyclovir for the patients with IC/BPS might have clinical efficacy. |
| | 6b | Bladder pain is the core symptom of IC/BPS, hence, the primary end point of this study is the improvement of bladder after the treatment |

| | | |
|---|---|---|
| Objectives | 7 | The visual analog scale (VAS) score for pain, quality of life score (QoL) based on urinary symptoms, Interstitial Cystitis Symptoms Index (ICSI), and Interstitial Cystitis Problem Index (ICPI). |

| | | |
|---|---|---|
| Trial design | 8 | A prospective study was conducted to evaluate to therapeutic effect of oral valacyclovir for the patients with IC/BPS. The diagnosis of IC/BPS was made through following the clinical symptom index of the American Urological Association guideline. The IC/BPS patients with concurrent urological diseases, such as neurogenic voiding dysfunction, ketamine cystitis or acute bacterial cystitis in recent one month would be excluded. The IC/BPS patients with possibility of pregnancy also would be rule out.<br><br>At first, the enrolled patients would be investigated for urinary analysis and culture to rule out current bacteriuria. The enrolled patients would receive a comprehensive medical history review. A pregnancy test would be performed for women of childbearing potential. The patients would be asked to provide urine sample to urinary virus investigation before the treatment. The IC/BPS patients would be investigated for baseline clinical symptoms questionnaire including VAS, QoL, ICPI, ICSI, and OSS. The parameters in the VUDS report also would be record. The Informed consent would be obtained from all individual participants before the intervention. Due to higher bioavailability, we would use valacyclovir in this study. The dose of valacyclovir we use is according to previous valacyclovir study for genital HSV infection. [18] According previous valacyclovir study for chronic recurrence herpesvirus infection disease, long-term therapy of oral valacyclovir < 1000mg/day should be effective and safe; the safety profiles of valacyclovir and placebo were similar. All of our patients would receive open-label oral valacyclovir 500 mg twice per day for 4 weeks.<br><br>We estimate to enroll 30 IC/BPS patients with evidence of EBV infection in bladder into this study. After the beginning of valacyclovir therapy, the patients would receive a telephone interview to evaluated current symptoms changes at first and second weeks. The overall improvement of current therapy would be assessed with Global Response Assessment (GRA) (3: symptoms free, 2: >50% symptoms improvement, 1: 25-50% symptoms improvement, 0: 0-25% symptoms improvement, -1: symptoms worse). The GRA, VAS, and any adverse effect would be investigated in the telephone interview. After the full 4 weeks treatment, the patients would be asked to back to our clinic. All symptoms questionnaire (GRA, VAS, QoL, ICPI, ICSI, and OSS) and adverse effect would be evaluated again. The patients would be asked to provide urine sample again. The primary endpoint of this study is the GRA at 4 weeks. The secondary endpoints include the changes of VAS, VAS, QoL, ICPI, ICSI, and OSS between baseline and end of the study. The following figure showed the study flow diagram. |

**Methods: Participants, interventions, and outcomes**

| | | |
|---|---|---|
| Study setting | 9 | This is a prospective phase I study which was conducted in a medical center Hualien Tzu Chi General Hospital in Taiwan. |
| Eligibility criteria | 10 | Inclusion Criteria:<br>patients with IC/BPS (according to American Urological Association guideline for IC/BPS 2015)<br>Exclusion Criteria:<br>patients with neurogenic bladder<br>patients with bladder outlet obstruction<br>patients with lymphoma<br>patients with bacteria cystitis |
| Interventions | 11a | oral valacyclovir 500 mg twice per day for 4 weeks |
| | 11b | The treatment would be stopped if the participant request due to any side effect or any reason. |
| | 11c | The patients were asked to follow up with telephone interview in the first week, second week and was followed in urologic clinic in the 4$^{th}$ week after the treatment. |
| | 11d | All medications which had been used by the participants before this study were allowed to keep using during this clinical trial. In the one month treatment course, additional pain control drugs, medications for urinary frequency, or any bladder procedures were prohibited. |
| Outcomes | 12 | The primary endpoint was the change in VAS pain score for bladder pain from the baseline to the end of week 4. Bladder pain is the core symptom of IC/BPS, hence, the primary end point of this study is the improvement of bladder after the treatment<br><br>The secondary end point included the changes of ICPI, ICSI and GRA. In addition, the patients were asked to provide urine sample for investigating urinary virus and the level inflammatory cytokines. |
| Participant timeline | 13 | |

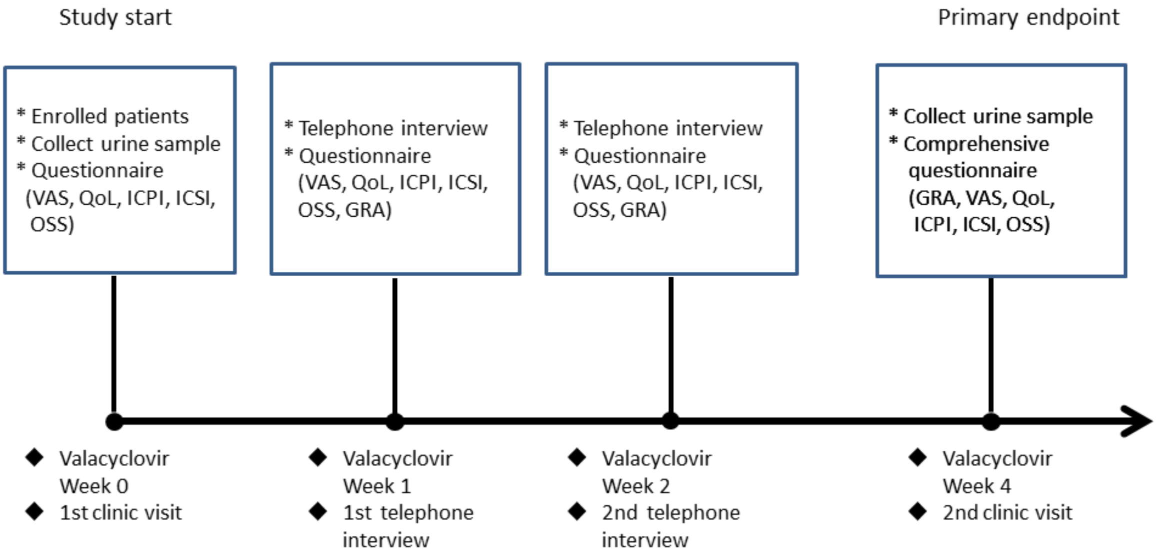

| | | |
|---|---|---|
| Sample size | 14 | We used G*Power 3.1.9.2 to calculate the sample size needed. For evaluating the change of VAS pain score of intervention in this before-after study, we set effect size of 0.6, α of 0.05, power(1-β) of 0.80, and two-sided test then got the estimated sample size 24. For estimating dropout rate 15%, we enrolled 28 patients with IC/BPS for the treatment. Finally all patients completed the study and were included into the final analysis |
| Recruitment | 15 | The patients with IC/BPS who were refractory to current treatments were asked to join our study in Dr. Kuo' and Dr. Jhang's clinic. |
| **Methods: Assignment of interventions (for controlled trials)** | | |
| Allocation: | | This is a phase I single arm study. |
| Sequence generation | 16a | Not applicable |
| Allocation concealment mechanism | 16b | Not applicable |
| Implementation | 16c | Not applicable |
| Blinding (masking) | 17a | Not applicable |
| | 17b | Not applicable |
| **Methods: Data collection, management, and analysis** | | |
| Data collection methods | 18a | After the patient were enrolled into this study, the baseline symptoms would be investigated with the commonly used questionnaires for IC/BPS, including VAS pain scale, ICSI, ICPI and GRA. The research assistant of this study project interviewed the patients in a meeting room near the urologic clinic for the baseline symptoms. The treatment results in the first week and 2nd week were obtained via telephone interview, and the patients were revisited Dr. Kuo's and Dr. Jhang's clinic at the end of the study and were interviewed in the meeting room for the final treatment results. |
| | 18b | We provided free medications valacyclovir for the patients and had telephone interview to ensure the patient could keep using the medication. |
| Data management | 19 | The questionnaires of each patients were collected and were created a profile. Research assistant key in the data to an Excel file. The paper questionnaire were storage in our office. Research Ethics Committee of Hualien Tzu Chi General Hospital oversaw the process and we provided a mid-term report the research ethics committee. |

| | | |
|---|---|---|
| Statistical methods | 20a | The clinical and laboratory results for each group were compared using nonparametric methods, including the Mann–Whitney U test for 2 nonpaired groups and the Wilcoxon matched-pair signed-rank test for 2 paired groups. A p value of <0.05 indicated a significant difference. All statistical analyses were performed using GraphPad Prism 8 (Boston, MA, USA). |
| | 20b | The patients were classified to responders and non-responders according to the VAS scale reduction $\geq$ 3 in the end of the study. |
| | 20c | All patients in this study completed the 4 weeks treatment and were interviewed for the questionnaire. All results were included for the final analysis. |

**Methods: Monitoring**

| | | |
|---|---|---|
| Data monitoring | 21a | This is pilot study which was only expected to enrol less than 30 patients, and the follow-up duration in this study is only one month. Ethics Committee of Hualien Tzu Chi General Hospital approved that data monitoring committee could be waived in this study. The data of this study was monitored by Ethics Committee of Hualien Tzu Chi General Hospital. |
| | 21b | The follow-up duration in this study is only one month and there is no interim analyses. Patients in this study were informed that they could stopped using the medication if they have any intolerable or any reason. We followed the patient at 1st, 2nd and the end of the study. |
| Harms | 22 | The patients were followed at 1st, 2nd and the end of the study. During the interview, the patients were asked if they had any unpleasant event after took the medication. |
| Auditing | 23 | Ethics Committee of Hualien Tzu Chi General Hospital inspected the process of this study and ensure the trial worked as the study project. |

**Ethics and dissemination**

| | | |
|---|---|---|
| Research ethics approval | 24 | The study project was submitted to Ethics Committee of Hualien Tzu Chi General Hospital in English and Chinese version, including the informed consent of this study. |
| Protocol amendments | 25 | Any modification of the protocol needed to be approved by Ethics Committee of Hualien Tzu Chi General Hospital, and any change of the inform consent also needed to be approved by Ethics Committee of Hualien Tzu Chi General Hospital. |

| | | |
|---|---|---|
| Consent or assent | 26a | The patients with IC/BPs who were compatible with inclusion and exclusion criteria were asked to join to this study by the principal investigator Dr. Jia-Fong Jhang and Dr. Hann-Chorng Kuo in his urology clinic. The researcher assistant explained the trial in detail and let the patient consider to join this trial or not. If the patients had any question for this trial, they could ask Dr. Kuo or Dr. Jhang again. If the patient agreed to joint the trial, the research assistant would give a inform consent to the patient and obtain the signature. |
| | 26b | In this study, we also asked the patients to provide their urine (50ml) at the baseline and the end of the study. Collection patients' urine specimens were notified in the inform consent and Ethics Committee of Hualien Tzu Chi General Hospital had approved the collection of the urine specimens. |
| Confidentiality | 27 | The personal information, results of the questionnaires and the inform consents were storage in the researcher office in Hualien Tzu Chi General Hospital. The data was only available to the research assistant and principal investigator Dr. Jhang. |
| Declaration of interests | 28 | This study was only funded by the Buddhist Tzu Chi Medical Foundation and the Ministry of Science and Technology of Taiwan government. The principal investigators did not have any financial support by pharmaceutical industry. |
| Access to data | 29 | The principal investigator Dr. Jhang had access to the final trial dataset. Co-PI Dr. Kuo also had access to the final trial dataset. The other hospital members did not have the access. |
| Ancillary and post-trial care | 30 | If patients had any discomfort after took valacyclovir, they would be back to our clinic for examination or medical treatment. Every participant of this study received 200 NTD fee at study beginning for the clinical trial enrolment. |
| Dissemination policy | 31a | This study did not have any industry sponsor, and the results were only completed by our research team. There is no nay publication restriction. |
| | 31b | The investigators who had contributed to this study were eligible to be the authors of this study, including patients enrolment, original idea, manuscript writing, supervision and consultation. The manuscript was written by the investigator Dr. Jia-Fong Jhang and had been sent for profession English editing. |
| | 31c | This protocol of this clinical trial had been registered in ClinicalTrials.gov and was public accessible. |

**Appendices**

| | | |
|---|---|---|
| Informed consent materials | 32 | The inform consent of this study was made according to the template which provided by Ethics Committee of Hualien Tzu Chi General Hospital, and the final version of the inform consent had been approved by the ethics committee. |
| Biological specimens | 33 | In this study, the patients were asked to provide urine sample at baseline and at the end of study. The urine samples were investigated for the urinary viral spectrum and the level of inflammatory cytokines. |

*It is strongly recommended that this checklist be read in conjunction with the SPIRIT 2013 Explanation & Elaboration for important clarification on the items. Amendments to the protocol should be tracked and dated. The SPIRIT checklist is copyrighted by the SPIRIT Group under the Creative Commons “Attribution-NonCommercial-NoDerivs 3.0 Unported” license.
