# Supplementary material for: Urinary Viral Spectrum in Patients with Interstitial Cystitis/Bladder Pain Syndrome and the Clinical Efficacy of Valacyclovir Treatment
Source: Biomedicines. 2024 Feb 26;12(3):522. doi: 10.3390/biomedicines12030522 (PMC10967905; doi:10.3390/biomedicines12030522)
Supplement: Supplementary file 1 [file biomedicines-12-00522-s001.zip › Supplementary Material Urinary Virus Detection.pdf]

Supplementary Material Urinary Virus Detection:

Urine samples of 50 mL were collected at baseline and 4 weeks after valacyclovir treatment. The samples were placed immediately on ice and then centrifuged at 1800× g for 10 minutes at 4°C. The supernatant was preserved in a freezer at −80°C. Before further analysis, the frozen urine samples were centrifuged at 12,000 × g for 15 minutes at 4°C, and the supernatants were used for subsequent experiments. The centrifuged supernatants (1 mL) were sent to the Medical Laboratory Department in Hualien Tzu Chi Hospital for urinary virus investigation. The urinary virus was EBV, BK virus (BKV), JCV, HSV, VZV, and cytomegalovirus (CMV).

The virus DNA polymerase chain reaction (PCR) protocol was developed in our laboratory department and passed the viral load survey offered by the College of American Pathologists. The urinary DNA extraction used the LabTurbo 48 compact system (LabTurbo, Taipei, Taiwan) with Virus Mini Kit (Catalog No: LVN480-1000; LabTurbo, Taipei, Taiwan).

First, proteinase K was added to the urine samples. Lysis of the sample was performed by adding lysis buffer VLL and incubating for 15 minutes at 57°C. We moved the sample to commercial column and then removed the extra buffer by ethanol and suction. The sample was washed with wash buffer LW1 and suctioned twice. Finally, elution buffer CCEB was added to elute the DNA/RNA extract, which was then ready for real-time PCR. The real-time PCR was performed with Rotor-Gene Q 5plex HRM (QIAGEN, Hilden, German) and the TaqMan system. The qPCR detection protocol was as follows:

1) For reaction mix in each well was concluding: oasig 10 ul, primer/probe mix 1 ul, and RNase/DNase free water 4 uL. Then, add 5 uL of DNA template into each reaction mix. The standard follows the manufacturer's instructions.
2) qPCR amplification takes 50 cycles, as follows: Enzyme activation at 95°C for 2 minutes, denaturation at 95°C for 10 seconds, and data collection at 60°C for 1 minute.

The EBV DNA detection was targeted on a nonglycosylated membrane protein (BNRF1) gene with a commercialized kit (Primerdesign, Chandler's Ford, UK). The BKV and JCV DNA detection was also performed with commercialized kits from Primerdesign. The BKV detection was targeted on a non-coding region, and the primers had 100% homology with more than 95% of reference sequences in the National Center for Biotechnology Information database; therefore, the quantification profile was very broad. The urinary VZV and HSV were investigated with another commercialized kit (LightMix, Roche, Basel, Switzerland, Cat.-No. 40-0358-96 and Cat.-No. 40-0562-32, respectively). We captured the results by reader.

Urinary cytokines investigations:

The level of urinary inflammatory cytokines and neurotrophins were investigated with commercial microspheres using the Milliplex Human cytokine/chemokine magnetic bead-based panel kit (Millipore, Darmstadt, Germany). The targets included interleukin (IL)-1β, IL-6, IL-8, IL-10, BDNF, tumor necrosis factor-α (TNF-α), monocyte chemoattractant protein-1 (MCP-1), and macrophage inflammatory protein-1 alpha (MIP-1a). The analytes were measured by using the multiplex kit (catalog number: HCYTMAG-60K-PX30). The procedures used to measure these urinary cytokines and chemokines were based on the manufacturer's instructions

and reported previously [2]. A total of 25-µL assay buffer, 25-µL urine sample, and 25-µL beads were sequentially added to 96-well plates (panel kits), and the plates were incubated overnight in the dark at 4°C. The contents of the wells were removed, and the plates were washed twice with 200-µL wash buffer. Then, 25 µL of detection antibody were added to each well, and the plates were incubated in the dark on a shaker plate for 1 hour at room temperature. Next, 25 µL of streptavidin/phycoerythrin solution was added into each well (to form a capture sandwich immunoassay); incubation was then performed in the dark for 30 minutes at room temperature. The well contents were again removed, and the plates were washed twice with 200-µL wash buffer. Finally, 150 µL of sheath fluid was added, and the plates were evaluated on the MAGPIX instrument with xPONENT software. Median fluorescence intensities of all cytokine/chemokine targets were analyzed to calculate the corresponding cytokine/chemokine concentrations in the urine samples. The detection powers of the biomarkers were provided by the manufacture: BDNF: 2.4-10,000 pg/mL, IL-1β: 0.5 - 25,000 pg/mL, IL-6:0.24 - 10,000 pg/mL, IL-8: 0.14 - 10,000 pg/mL, L-10: 0.7 - 40,000 pg/mL, TNF-α: 1.2 - 100,000 pg/ml, MCP-1: 3 - 50,000 pg/mL, MIP-1α: 0.3 - 50,000/mL.
